# Lipid-Lowering Treatment and the Lipid Goals Attainment in Patients with a Very High Cardiovascular Risk

**DOI:** 10.3390/jcdd10080329

**Published:** 2023-08-02

**Authors:** Anna Lis, Paulina Lis, Weronika Łowicka, Małgorzata Grabarczyk, Michał Wita, Piotr Żarczyński, Małgorzata Żarczyńska, Maciej Haberka

**Affiliations:** 1 Cardiology Students’ Scientific Association, Department of Cardiology, SHS, Medical University of Silesia, 40-635 Katowice, Poland; 2 Health Promotion and Obesity Management Unit, Department of Pathophysiology, Faculty of Medicine in Katowice, Medical University of Silesia, 40-752 Katowice, Poland; 3 Department of Cardiology, SHS, Medical University of Silesia, 40-055 Katowice, Poland

**Keywords:** hypercholesterolemia, lipid goals, cardiovascular risk, LDL, high-dose statin

## Abstract

Hypercholesterolemia is the main cardiovascular (CV) risk factor with a large body of evidence. Our aim was to assess the achievement of the main therapeutic goal of Low-Density Lipoprotein Cholesterol (LDL-C) in patients with a very high CV risk and a high-dose statin therapy. The study group consisted of 1413 consecutive patients hospitalised at the Upper-Silesian Medical Centre in Katowice due to acute myocardial infarction (AMI) treated with atorvastatin ≥ 40 mg or rosuvastatin ≥ 20 mg. The lipid profile was performed on admission and within 12 months after AMI. The main therapeutic goal was defined as LDL-C < 55 mg%. The study group (*n* = 1413) included 979 males (69.3%) with arterial hypertension (83.3%), diabetes (33.5%), peripheral artery disease (13.6%) and nicotinism (46.2%). In the study group, only 61 patients (4.3%) were additionally taking ezetimibe. During hospitalisation, the primary LDL-C goal was found in only 186 patients (13.2%). Subsequently, a follow-up lipidogram within 12 months was performed in 652 patients (46%), and the therapeutic goal was achieved in 255 patients (39%). There were 258 (18.26%) patients who died within 12 months after myocardial infarction. The lowest mortality rate was found in the subgroup of patients with LDL-C < 55 mg% during follow-up (11.02%). The primary lipid goal attainment among patients with a high-dose statin and a very high CV risk is low and far from the expected rate. Patients hospitalised for AMI should be given a combination of statin and ezetimibe more frequently. Low LDL-C levels measured at follow-up predict a lower risk of death at 12-month follow-up in a large group of patients.

## 1. Introduction

Cardiovascular (CV) disease consistently ranks as the number one cause of death in Poland and Europe. Of these, ischaemic heart disease and cerebrovascular diseases are the most common contributors to mortality [1]. Coronary artery disease (CAD) leads to a higher risk of CV events [2]. There are many risk factors for CV disease. Among the modifiable ones, poor dietary habits, insufficient physical activity and lipid disorders are the most prevalent and important [3]. Hypercholesterolemia is one of the well-evidenced and frequent risk factors [4]. Studies showed that LDL cholesterol (LDL-C)-lowering therapies are associated with reduced risk of CV death, myocardial infarction (MI) or other acute coronary syndrome (ACS) [5]. The European Society of Cardiology (ESC) guidelines recognise LDL-C lowering as the key to CV prevention [6]. High-intensity statin therapy was shown to reduce the risk of atherosclerotic CV disease in both primary and secondary prevention [7]. Statins reduce the rate of cholesterol biosynthesis, which contributes to the increased expression of the LDL receptor (LDL-R). This results in the increased uptake of LDL-C particles from the bloodstream, thus reducing its blood concentration. The degree of reduction in LDL-C concentration is dose-dependent. An intensive statin therapy defined as atorvastatin ≥ 40 mg or rosuvastatin ≥ 20 mg is expected to reduce LDL-C by ≥50% [8,9]. Hypolipidemic treatment with statins is recommended according to the principles “the lower, the better” and “the earlier, the better” [10]. Unfortunately, bad opinions about statin treatment have become widespread, and have contributed to a growing reluctance of patients towards this group of drugs, resulting in increasing incompliance and premature discontinuation of their use [11].

The primary aim of this study was to assess the achievement of the main therapeutic goal of LDL-C among patients with a very high CV risk and high-dose statin therapy. The secondary aim was to assess the rate of death from CV causes within 12 months after myocardial infarction.

## 2. Materials and Methods

This was a retrospective, cross-sectional study performed on patients treated in the Upper-Silesian Medical Centre in Katowice. All the consecutive patients (*n* = 30,000) admitted to the hospital (January 2019 to December 2021) were screened and the final study included 1413 patients (979 males; 69.3%; 66 ± 11.56 years old) admitted with acute myocardial infarction (AMI).

The inclusion criteria were: hospitalisation in our medical centre with a diagnosis of AMI-STEMI or NSTEMI and a high dose of statin defined as atorvastatin ≥ 40 mg or rosuvastatin ≥ 20 mg. The exclusion criteria were: death during hospitalisation and a history of statin intolerance or known familial hypercholesterolemia. The complete lipidogram was checked in all admitted patients on the first day of hospitalisation. The LDL-C concentration was determined using the Friedewald formula at admission and during the long-term outpatient follow-up between 6 and 12 months after hospitalisation, to assess the primary therapeutic goal attainment: LDL-C < 55 mg% for secondary prevention. According to the European Society of Cardiology/European Atherosclerosis Society (ESC/EAS) guidelines for the management of dyslipidaemias, patients with previous ACS belong to a very high CV risk group. Patients classified as being at the highest CV risk should achieve a LDL-C level < 55 mg% and a reduction in initial concentration of at least 50% [12]. The MI was defined according to the ESC recommendations based on the fourth universal definition of myocardial infarction [13]. The obtained data for clinical characteristics included: CAD, arterial hypertension (HA), history of STEMI or NSTEMI, unstable angina (UA), previous coronary invasive procedures—percutaneous coronary intervention (PCI) or coronary artery bypass graft surgery (CABG), stroke, renal failure, estimated glomerular filtration rate (eGFR) value, type 1 or type 2 diabetes mellitus (DM), atherosclerotic disease of the peripheral arteries (PAD) or a history of nicotinism. The CAD was defined as a pathological process involving the formation of atherosclerotic plaques in the epicardial arteries, which may or may not lead to their narrowing and/or closure evidenced in either invasive or non-invasive imaging methods [14]. HA was classified according to the ESC 2021 guidelines for CV disease prevention in clinical practice [6]. Stroke was characterised as a neurological deficit attributed to an acute focal injury of the central nervous system (CNS) by a vascular cause, including cerebral infarction, intracerebral haemorrhage (ICH) and subarachnoid haemorrhage (SAH), according to an updated definition of stroke for the 21st century by American Heart Association/American Stroke Association 2013, modified in 2021 [15]. Renal failure was defined as eGFR < 60 mL/1.73 m^2^/min [16]. DM was classified in accordance with the clinical recommendations for the management of patients with diabetes 2022—position paper of the Polish Diabetological Association [17]. PAD was defined as a stenosis or occlusion of upper- or lower-extremity arteries, in line with American Heart Association’s Atherosclerotic Peripheral Vascular Disease Symposium II [18]. The clinical characteristics were based on the available medical records and the index hospitalisation.

### 2.1. Follow-Up

All the patients were followed on an outpatient basis for three years, starting on the day of discharge. The National Health System data were used to obtain the following data: death and hospitalisations with the clinical reason. The LDL-C was available during the follow-up period in 652 (46.14%) of the study patients.

The research was conducted in accordance with the Helsinki Declaration.

### 2.2. Statistical Analysis

All the results presented in the text, tables and figures are expressed as means ± standard deviation (SD) or number and percentage. The data normal distribution was analysed with the Kolmogorov–Smirnov test. The rates of patients within the category of LDL-C were compared using Chi-squared test. A value *p* < 0.05 was considered statistically significant. Statistical analysis was undertaken using MedCalc software (Version 20.218).

### 2.3. Limitations of the Study

The main limitation of our study is that the LDL-C was not available in the follow-up period in all the recruited study patients. Moreover, we are not aware of the hypolipidemic treatment prior to the index hospitalisation. Furthermore, this is a retrospective monocentric registry.

## 3. Results

A total of 1413 patients were enrolled in the study with a mean age of 66 ± 11.56 years; 69.29% were male. Of the patients who were in the study group, 83.30% had confirmed HA and 46.50% had ischaemic heart disease. Patients with diabetes were also a significant part, accounting for 33.55% of the study group. A similar proportion were those with chronic kidney disease (CKD)—29.16%. Of the study group, 13.59% had a history of peripheral arterial disease and 8.99% a history of stroke. A positive history of ACS with ST elevation was present in 16% of the study group, non-ST elevation MI in 17.83% and UA in 5.10%. Before hospitalisation, 30.01% of the subjects had undergone percutaneous coronary angioplasty and 13.87% CABG. Nicotinism was reported by 46.21% of the patients (Table 1).

**Figure 1:**
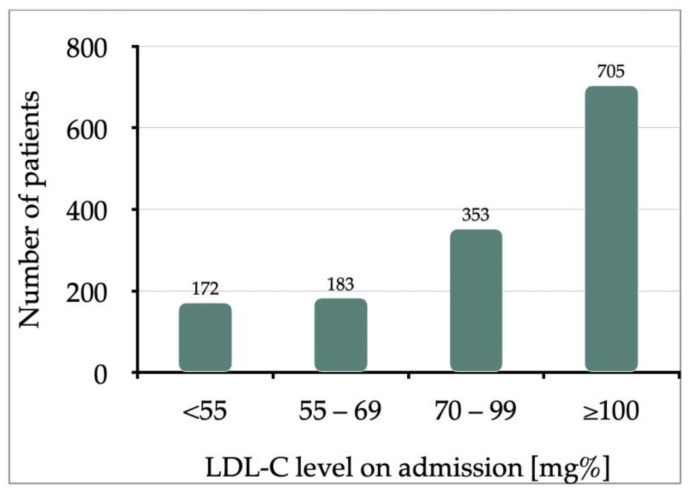
Low-Density Lipoprotein Cholesterol (LDL-C) levels measured in study patients on admission (*n* = 1413 patients); *p*-value as follows: *p* = 0.52, *p* < 0.0001, *p* < 0.0001.

At the time of admission, among 1413 patients, 172 (12.17%) had LDL-C < 55 mg%, 183 (12.95%) had LDL-C between 55 and 69 mg%, 353 (24.98%) had LDL-C between 70 and 99 mg%, and 705 (49.89%) had LDL-C ≥ 100 mg% (Figure 1).

During the observation period, 412 (29.16%) patients were hospitalised at least once again for CV reasons. The main reasons for readmission to the hospital were: atherosclerotic CV disease (125 cases, 30.34%), AMI (76 cases, 18.45%) and congestive heart failure (44 cases, 10.68%). During follow-up, 258 (18.26%) patients died.

Sixty-one (4.32%) patients received combination therapy with statin and ezetimibe. Ten (16.39%) of them died during the follow-up period, whereas sixteen (26.23%) of them achieved a therapeutic goal of LDL-C < 55 mg%.

### Follow-Up

**Figure 2:**
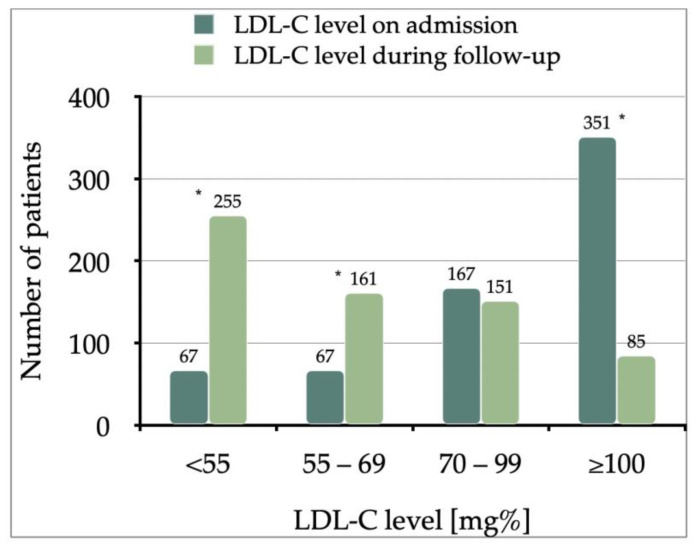
Comparison of Low-Density Lipoprotein Cholesterol (LDL-C) levels on admission and during follow-up period (*n* = 652 patients). * *p* < 0.0001; remaining: *p* = 0.3.

All the patients with available LDL-C in the next 6 to 12 months after the index MI were included in the follow-up—a subgroup of 652 patients (46.14%), and 255 (39.11%) of them achieved a therapeutic goal of LDL-C < 55 mg% (Figure 2).

Among those who had achieved the therapeutic goal of LDL-C < 55 mg% during the follow-up period, 28 patients (11.02%) died.

## 4. Discussion

The primary aim of this study was to assess the achievement of the main therapeutic goal of LDL-C among patients with a very high CV risk and high-dose statin therapy. The secondary aim was to assess the rate of death from CV causes within 12 months after MI.

CV diseases are one of the most common causes of death and disability [19]. According to the current guidelines, patients with a history of MI should aim at a target LDL-C level < 55 mg% to reduce the risk of death [9]. Our study assessed the attainment of the primary therapeutic goals in patients at very high CV risk by comparing LDL-C levels at the time of admission to the hospital and in the follow-up. On admission to the hospital, 185 out of 1413 patients (13.09%) had an LDL-C < 55 mg%, while the rest had higher LDL-C levels. Studies show that even at discharge after hospitalisation for ACS, not all patients receive intensive statin therapy and, despite available treatments, lipid management of very high CV risk patients is sub-optimal [20]. During our follow up, LDL-C concentrations were available in 652 (46.14%) patients. Despite intensive statin treatment, only 255 (39.11%) of them managed to achieve the therapeutic goal of LDL-C < 55 mg%. These people had the lowest risk of death during follow-up. However, only 61 (4.32%) patients received combined therapy, including statin and ezetimibe. In this group, 16 (26.23%) people achieved the intended LDL-C level, and death occurred in 10 (16.39%) individuals.

One of the studies that also assesses the possibility of achieving the therapeutic goal in terms of LDL-C is the analysis based on the POLASPIRE registry, which included 1026 patients hospitalised for ACS, UA, scheduled PCI or CABG. The study group was divided into two subgroups with (DM+, 332 patients) and without diagnosed DM (DM–, 694 patients). The control showed that patients with DM had significantly higher systolic blood pressure (SBP), body mass index (BMI), waist circumference (WC), triglycerides(TG) and lower LDL-C and HDL-C levels compared to people without DM [2,21]. Recommendations for statin therapy were similar for both groups. Despite the very high CV risk among all patients, rosuvastatin was used in only one-fifth of patients, despite its more beneficial effect on lowering LDL levels [22], and atorvastatin was the most popular lipid-lowering drug. The prevalence of ezetimibe use was also very low in both subgroups. The objectives of the study were based on the 2016 guidelines; when it comes to the LDL level, it was LDL < 70 mg%. The results of the study showed that the percentage of achievement of the primary goal (LDL: 51% vs. 35%; *p* < 0.0001) was higher in the group of DM+ than in the group of DM− patients [2,21].

In turn, the analysis based on the “EUROASPIRE V” registry showed that only 30% of patients achieved the LDL-C level < 70 mg% 12 months after discharge from the hospital after AMI [23]. A Baigent meta-analysis of nearly 170,000 patients, most of whom had documented ischemic heart disease, comparing 26 studies, showed that each 1.0 mmol/L (≈39 mg%) reduction in LDL-C results in a 20% relative reduction in annual adverse event rate, including coronary death, non-fatal MI, coronary revascularization and ischemic stroke [24].

Despite the differences in therapeutic goals (LDL-C ≤ 55 vs. < 70 mg%), less than half of patients achieve the therapeutic goal in both our and the cited studies. Note that patients received the maximum tolerated dose, not the maximum available. We also have no information on lifestyle modifications by patients. It is also worth pointing out that despite the failure to achieve the desirable therapeutic goal, treatment combined with statins and ezetimibe was received by a very small group of patients. This is a problem because, according to current knowledge, the use of combined therapy with these drugs can be beneficial [9,25].

The cited studies are based on different guidelines regarding the therapeutic goal in terms of LDL-C level. Also worth mentioning is the DA VINCI study comparing recommendations for target LDL-C. The 2018 American College of Cardiology/American Heart Association (ACC/AHA) guidelines differ from the 2019 European Society of Cardiology/European Atherosclerosis Society (ESC/EAS) guidelines for patients with atherosclerotic cardiovascular disease (ASCVD) (<70 vs. <55 mg%, respectively). Relative and absolute risk reduction (RRR and ARR) were simulated. Among patients with ASCVD, achieving the LDL-C ESC/EAS target may result in an additional ARR of 2% over 10 years compared to the ACC/AHA approach [26].

Statins are drugs used alone or in combination with ezetimibe, which inhibits the absorption of cholesterol in the intestines [27]. This combination of drugs is very beneficial because, when statins lower lipids by reducing endogenous cholesterol production in the liver, the body responds by boosting cholesterol absorption, which can reduce statin efficacy. As a result, the addition of ezetimibe can give extra benefit by limiting cholesterol absorption, enhancing the efficacy of statins to lower LDL-C [28]. Such therapies are used because atherosclerosis is the most common process responsible for MI, stroke and peripheral vascular diseases, and therefore leads to a significant reduction in life expectancy and quality of life [29]. Unfortunately, despite widely available evidence on the effectiveness of statin use [30] to lower LDL-C in this group of patients, many of them fail to achieve the appropriate therapeutic goal.

Despite early revascularization, LDL-C lowering has a well-documented prognostic effect in secondary CV disease prevention [31]. Unfortunately, even with combined therapy with statins and ezetimibe, many patients fail to achieve their therapeutic goals. It is, therefore, worth considering the possibility of using another group of drugs, which are inhibitors of the protein convertase subtilisin/kexin type 9 (PCSK9). The mechanism of action of these drugs is to block the binding of PCSK9 to LDL receptors on the surface of hepatocytes. This, in turn, significantly reduces the degradation of LDL receptors in lysosomes, enables their recirculation to the cell membrane and has a positive effect on the removal of LDL-C from the plasma [32]. Two monoclonal antibodies, evolocumab and alirocumab, deserve special attention [33]. It has been proven that the combination of statins and PCSK9 inhibitors results in extremely low LDL-C concentrations and a reduction in CV events in secondary prevention [34]. In combination with statins in maximum tolerated doses, alirocumab and evolocumab reduced LDL-C levels by 46.73% more than placebo and by 30% more than ezetimibe [35]. Summarising all the above reports, it should be mentioned that in very high risk patients who are unlikely to achieve their therapeutic goal with statins, the method of first choice in lowering LDL-C should be a combination of statins and ezetimibe. If there is no improvement (reduction < 50% or LDL-C > 1.4 mmol/L), adding a PCSK9 inhibitor should be considered. PCSK9 inhibitors can be used in treatment while patients have statin intolerance [36]. In case of extreme risk (previous cardiovascular events or multi-vessel disease, familial hypercholesterolemia), starting therapy straight away with statins, ezetimibe and a PCSK9 inhibitor may be considered [25,37]. The results of studies that evaluate the effectiveness of new drugs, such as inclisiran, PPARβ/δ agonists or liver X receptor agonists, in reducing LDL-C levels and CVD risk, should be monitored [38,39].

Overall, our study is consistent with other reports and shows that few patients achieve treatment goals that significantly reduce CV disease risk. Despite this, few people receive maximum doses of drugs and therapy combined with ezetimibe or PCSK9 inhibitors. It is, therefore, worth trying to further increase the number of people reaching the target level of LDL-C, using available medicines.

## 5. Conclusions

The primary lipid goal attainment among patients with a high-dose statin and a very high CV risk is low and far from the expected rate. Patients hospitalised for AMI should be given a combination of statin and ezetimibe more frequently—this study suggests the potential benefit of combined therapy with statins and ezetimibe in achieving LDL-C goals. Low LDL-C levels measured at follow-up predict a lower risk of death at 12-month follow-up in a large group of patients. This study demonstrates the continuing need to optimise lipid-lowering therapy to achieve treatment goals and reduce cardiovascular risk in patients with a history of AMI. It is advisable to perform further population studies addressing this pressing issue in the cohort of patients after myocardial infarction treated with effective pharmacotherapy.

## Figures and Tables

**Table 1:** Characteristics of the study group.

Value (Mean ± SD) or *n* (%)	Parameter
66 ± 11.56	Age, years
979 (69.29)	Males
434 (30.71)	Females
657 (46.50)	Coronary heart disease
1177 (83.30)	Arterial hypertension
474 (33.55)	Diabetes mellitus
192 (13.59)	Peripheral arterial disease
127 (8.99)	History of stroke
	Chronic kidney disease
412 (29.16)	eGFR ^1^ < 60 mL/kg/1.73 m^2^
653 (46.21)	Smoking
	History of myocardial infarction
226 (16)	STEMI ^2^
252 (17.83)	NSTEMI ^3^
72 (5.10)	History of unstable angina
424 (30.01)	History of PCI ^4^
196 (13.87)	History of CABG ^5^

^1^ eGFR—estimated glomerular filtration rate; ^2^ STEMI—ST-elevation myocardial infarction; ^3^ NSTEMI—non-ST-elevation myocardial infarction; ^4^ PCI—percutaneous coronary interventions; ^5^ CABG—coronary artery bypass grafting.

## Data Availability

Data available upon a reasonable request.
